# Supplementary material for: Antibacterial Activity and Cytocompatibility of Electrospun PLGA Scaffolds Surface-Modified by Pulsed DC Magnetron Co-Sputtering of Copper and Titanium
Source: Pharmaceutics. 2023 Mar 14;15(3):939. doi: 10.3390/pharmaceutics15030939 (PMC10058054; doi:10.3390/pharmaceutics15030939)
Supplement: Supplementary file 1 [file pharmaceutics-15-00939-s001.zip › pharmaceutics-2248041-supplementary.pdf]

pharmaceutics

Article Support Information

Support Information of: Antibacterial Activity and Cytocompatibility of Electrospun PLGA Scaffolds Surface-Modified by Pulsed DC Magnetron Co-Sputtering of Copper and Titanium

Arsalan D. Badaraev [1], Marat I. Lerner [2], Olga V. Bakina [2], Dmitrii V. Sidelev [1], Tuan-Hoang Tran [1], Maksim G. Krinitcyn [1,2], Anna B. Malashicheva [3], Elena G. Cherempey [1], Galina B. Slepchenko [1], Anna I. Kozelskaya [1], Sven Rutkowski [1*], Sergei I. Tverdokhlebov [1*]

[1] National Research Tomsk Polytechnic University, 30 Lenin Avenue, 634050 Tomsk, Russia
[2] Institute of Strength Physics and Materials Sciences of Siberian Branch of the Russian Academy of Sciences, 2/4 Akademicheskii Avenue, 634055 Tomsk, Russia
[3] Institute of Cytology RAS, 4 Tikhoretsky Avenue, 194064 Saint Petersburg, Russia
* Correspondence: rutkowski_s@tpu.ru (S.R.); tverd@tpu.ru (S.I.T.)

1. Supplementary Figures

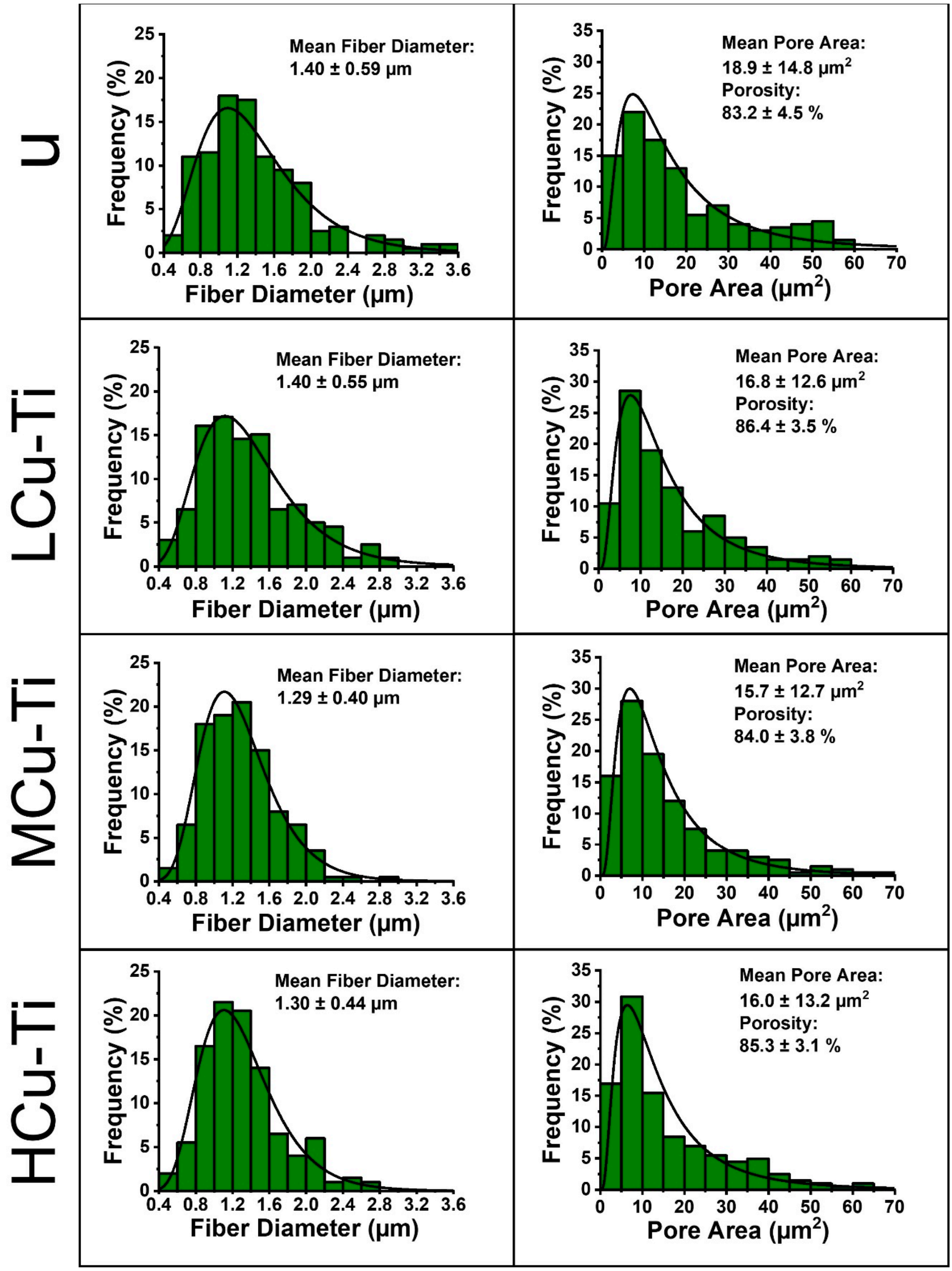

Figure S1. Fiber diameters and pore area histograms with porosity values of unmodified (u) and surface-modified by copper-titanium (Cu-Ti) thin films with different copper amounts (LCu-Ti, MCu-Ti, HCu-Ti) scaffolds from poly(lactide-co-glycolide) (PLGA): a) fiber diameter histograms with mean fiber diameter values, b) pore area histograms with mean pore area and mean porosity values.

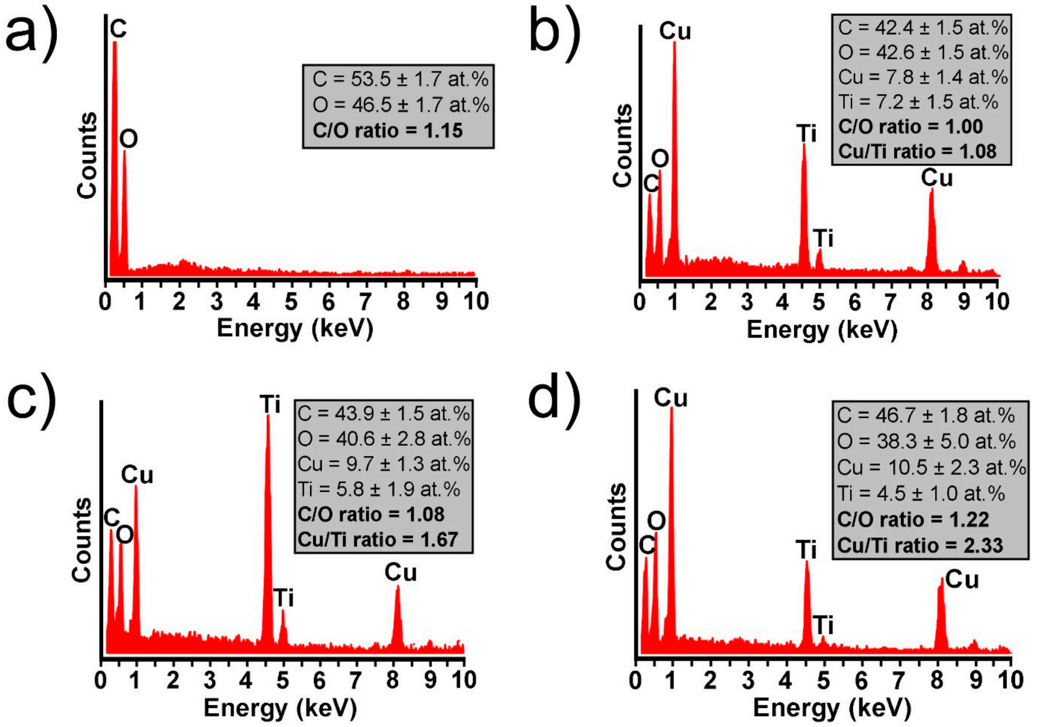

Figure S2. EDX spectra of unmodified (u) and surface-modified by copper-titanium (Cu-Ti) thin films with different copper amounts (LCu-Ti, MCu-Ti, HCu-Ti) scaffolds from poly(lactide-co-glycolide) (PLGA): (**a**) u, (**b**) LCu-Ti, (**c**) MCu-Ti, (**d**) HCu-Ti. All elemental concentration values are presented in atomic percent (at.%).

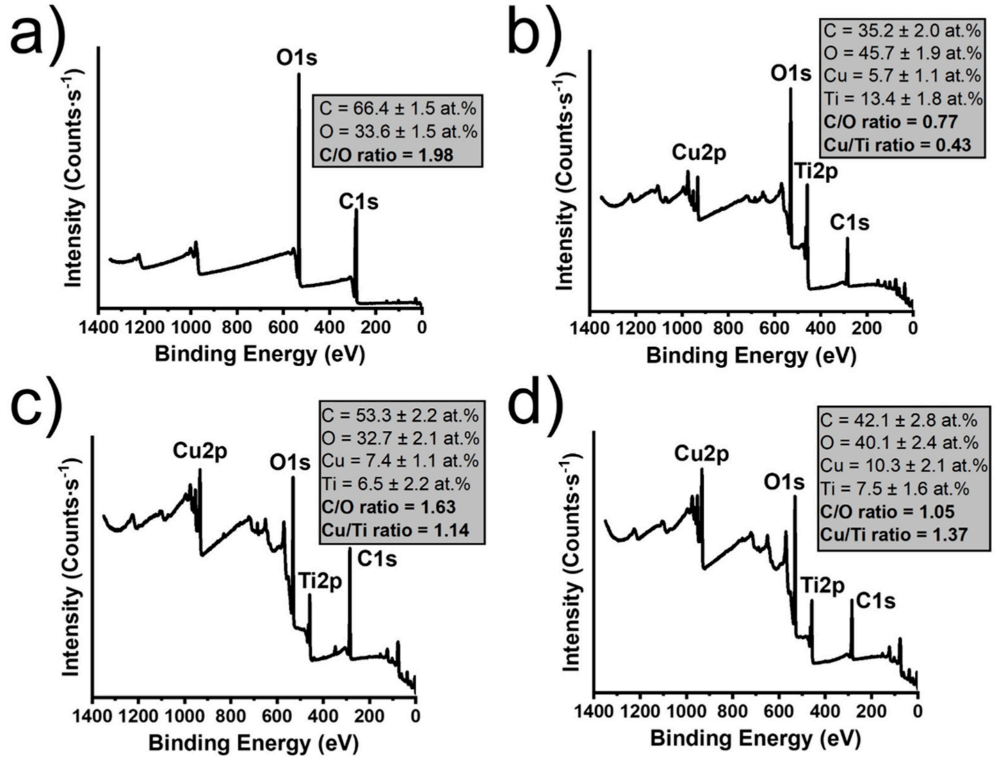

Figure S3. XPS survey spectra of unmodified (u) and surface-modified by copper-titanium (Cu-Ti) thin films with different copper amounts (LCu-Ti, MCu-Ti, HCu-Ti) scaffolds from poly(lactide-co-

glycolide) (PLGA): (a) u, (b) LCu-Ti, (c) MCu-Ti, (d) HCu-Ti. All elemental concentration values are presented in atomic percent (at.%).

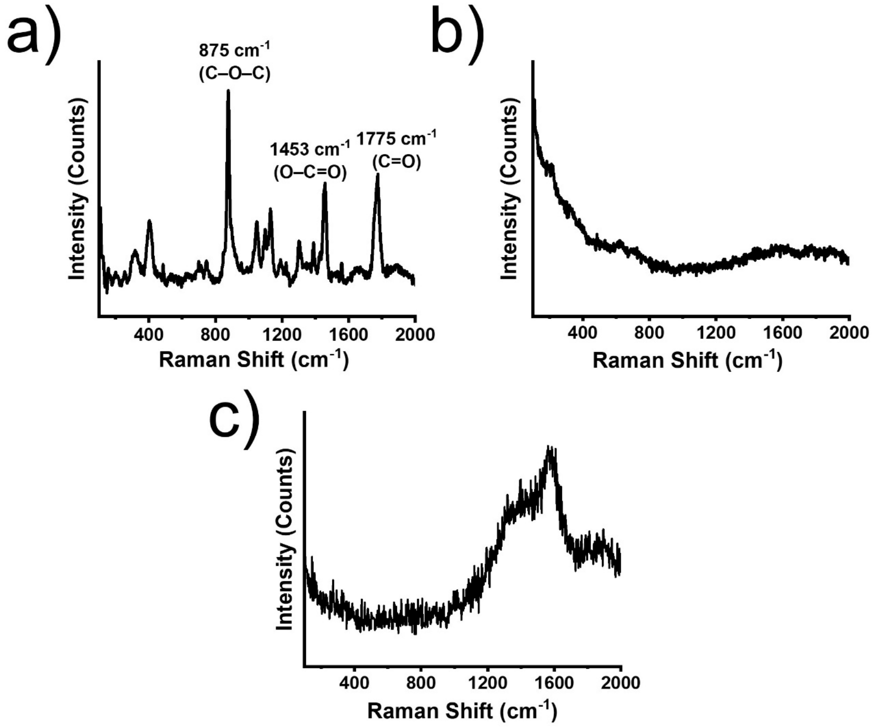

Figure S4. Raman spectra of unmodified (u) surface-modified by copper-titanium (Cu-Ti) thin films (MCu-Ti) scaffolds from poly(lactide-co-glycolide) (PLGA): (a) u, (**b**) MCu-Ti before treatment, (**c**) MCu-Ti after treatment.

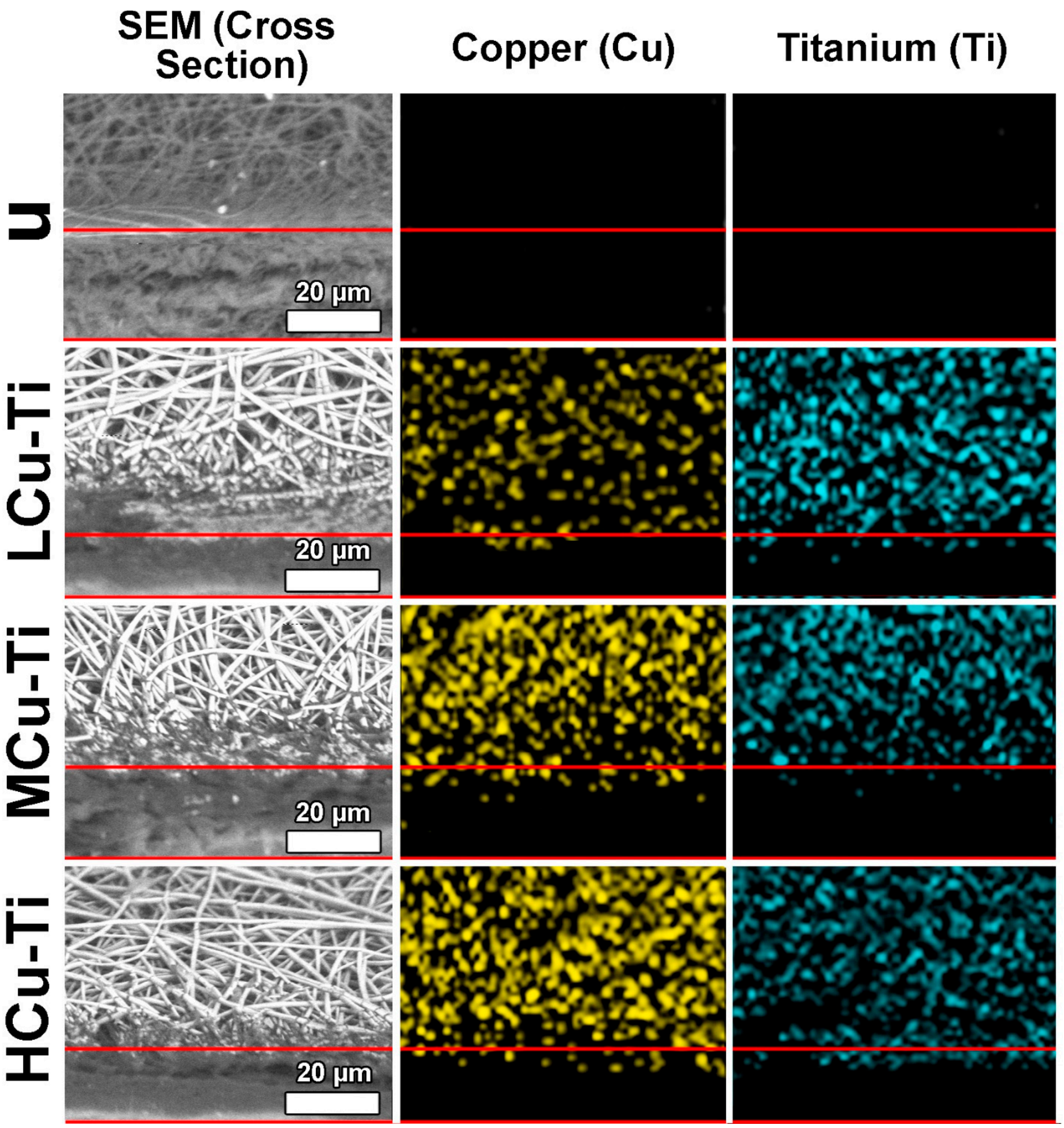

Figure S5. Cross sectional SEM micrographs (left column) and EDX mapping micrographs of the elements copper (Cu in column in the middle) and titanium (Ti in the right column) shows their distribution in the PLGA samples. In each SEM image above the upper red line show the polymer fibers located on the surface of the PLGA scaffolds. Below the upper red line are the polymer fibers, which are located inside of PLGA scaffolds volume. Images and EDX mapping were obtained at 2500× magnification.

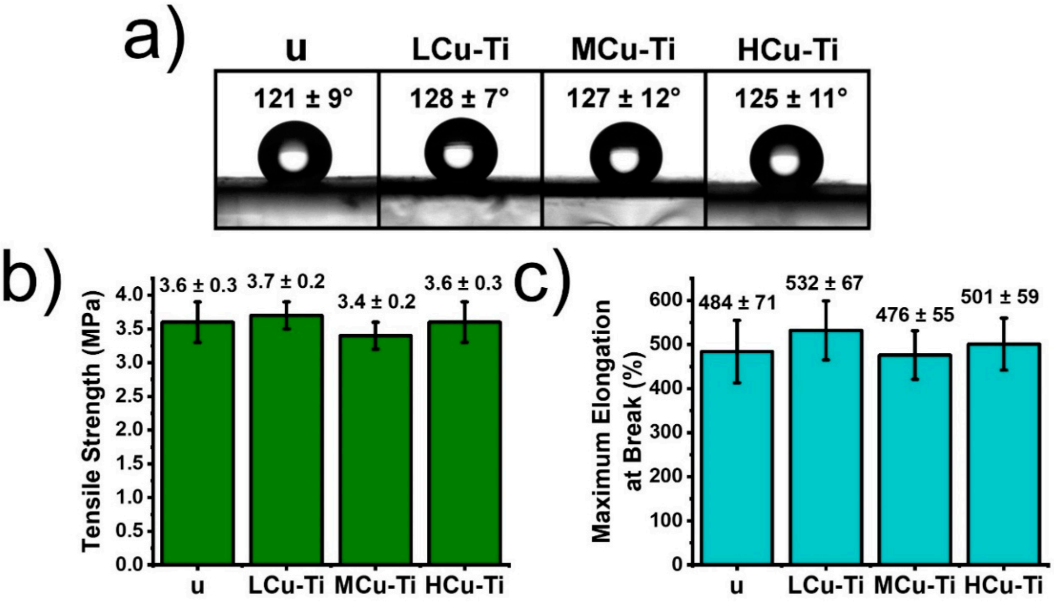

Figure S6. (**a**) Water contact angles (WCA), (**b**) mechanical characterization via tensile strength and (**c**) maximum elongation at break of unmodified (u) and surface-modified with Cu-Ti thin films (LCu-Ti, MCu-Ti, HCu-Ti) scaffolds from poly(lactide-co-glycolide) (PLGA).

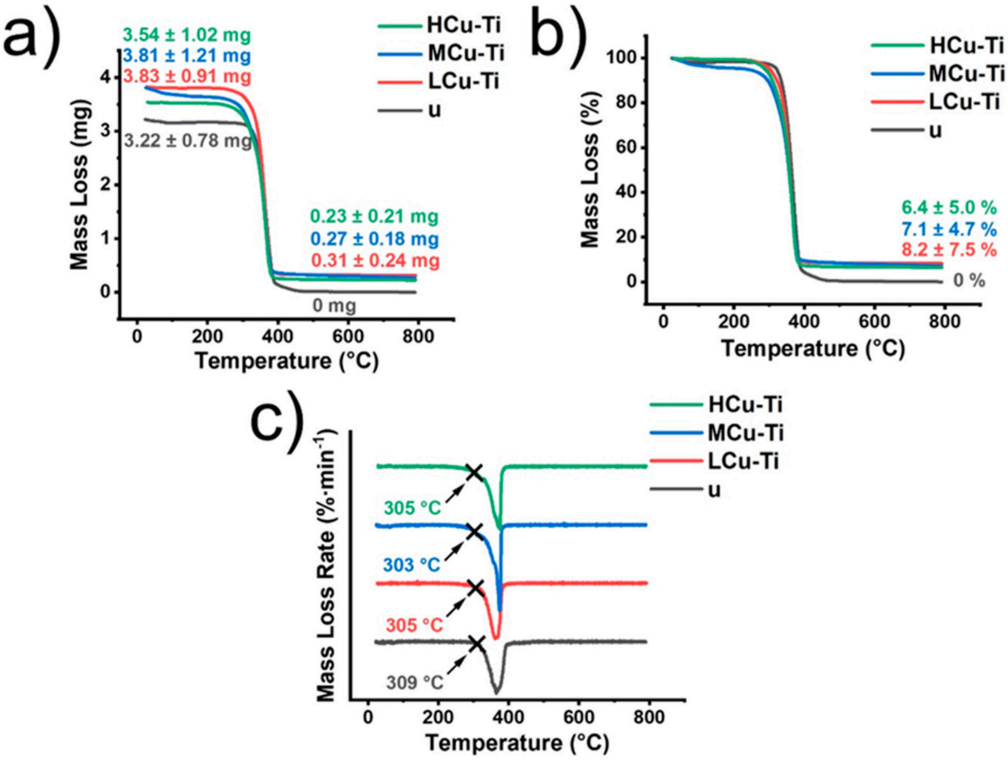

Figure S7. Thermal gravimetric (TG) and differential thermogravimetric (DTG) curves of unmodified (u) and surface-modified with Cu-Ti thin films (LCu-Ti, MCu-Ti, HCu-Ti) scaffolds from poly(lactide-co-glycolide) (PLGA): (**a**) TG curves with OY axis in milligrams (mg), (**b**) TG curves with OY axis in percent's (%), (**c**) DTG curves.

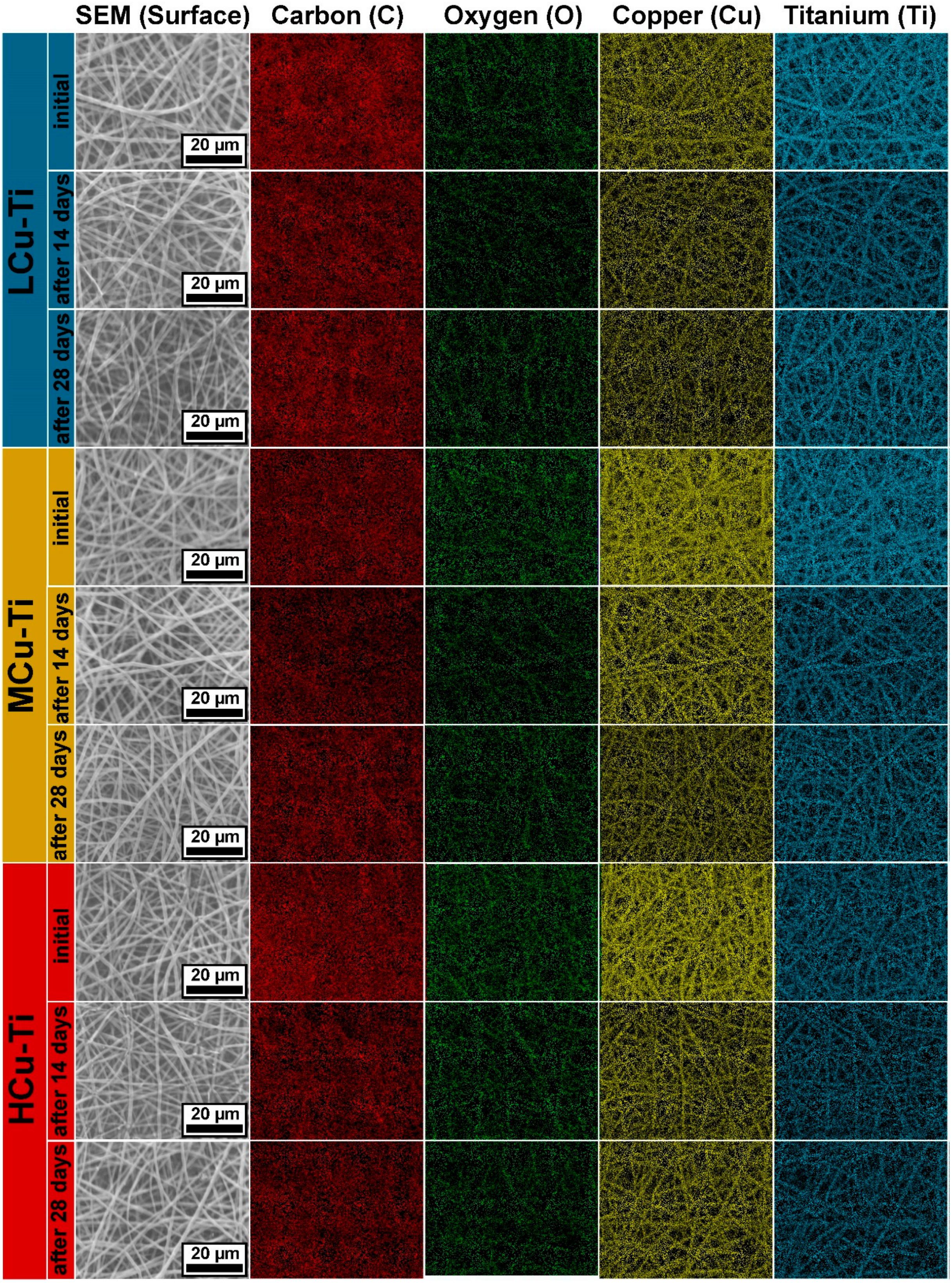

Figure S8. SEM micrographs of PLGA scaffold surfaces to the left and EDX mapping micrographs of the elements carbon (C in the second column), oxygen (O in the third column), copper (Cu in the fourth column) and titanium (Ti in the fifth column). Images and EDX mapping were obtained at 5000× magnification. The scale bars in the left column are representative of all micrographs.

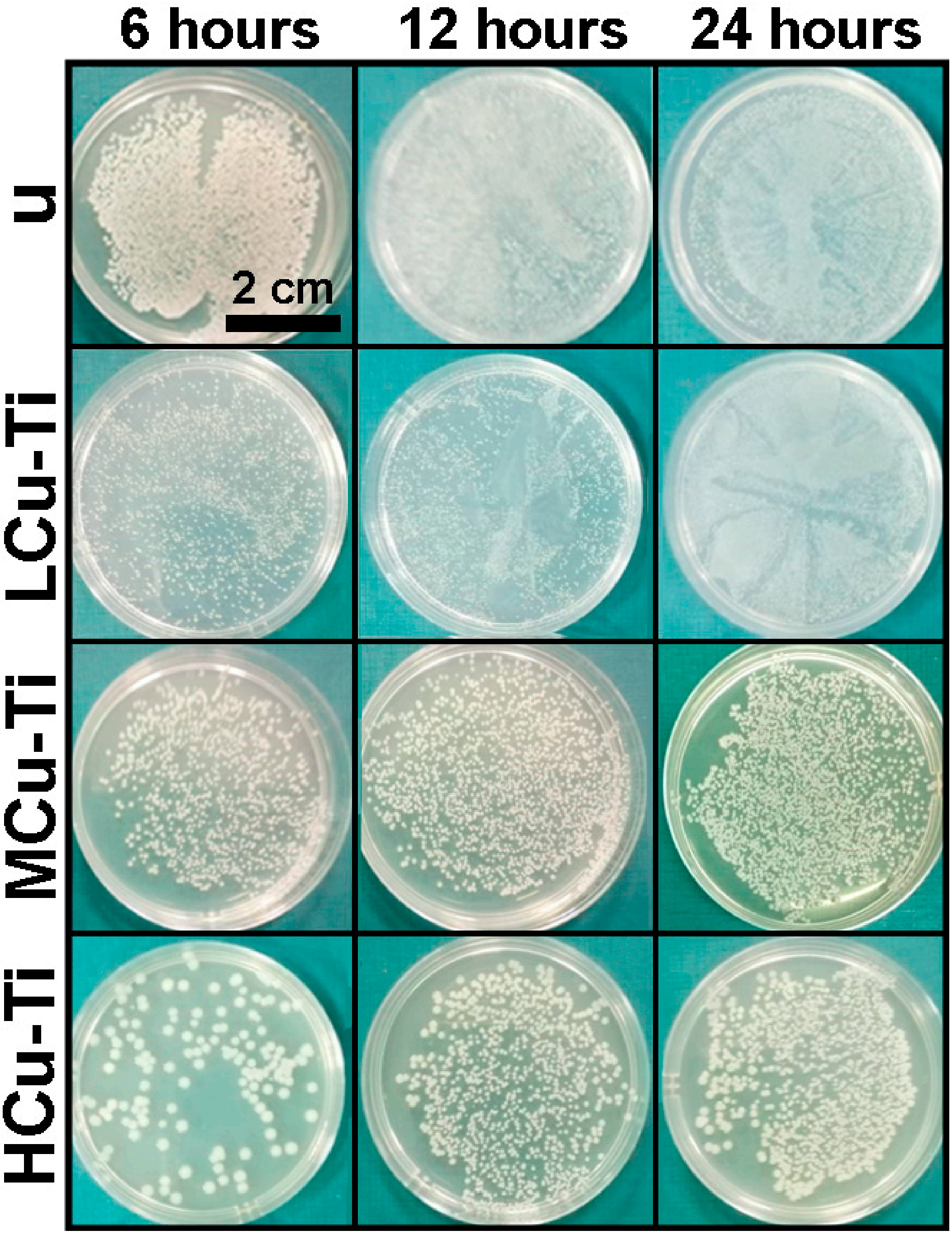

Figure S9. Photographs of Petri dishes with colonies of methicillin-resistant bacterium Staphylococcus aureus (MRSA) for unmodified (u) and surface-modified with a Cu-Ti thin films (LCu-Ti, MCu-Ti, HCu-Ti) scaffolds from poly(lactide-co-glycolide) (PLGA). Incubation times of 6 hours (left column), 12 hours (column in the middle) and 24 hours (right column) were performed in tubes with PLGA scaffold sample and bacterial suspension.

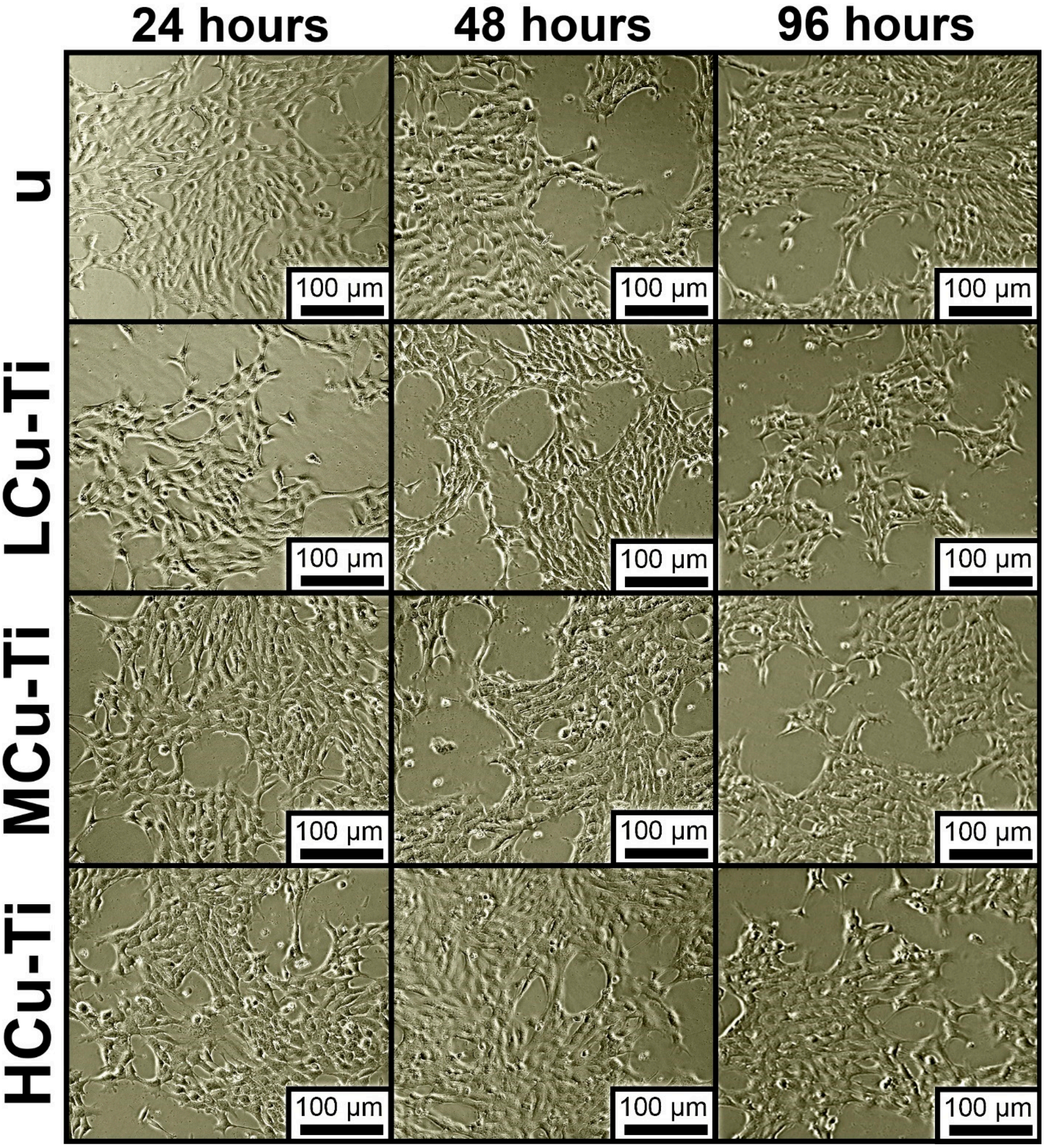

Figure S10. Optical micrographs of NIH/3T3 cells at 500× magnification incubated with non-diluted PLGA sample extracts (u, LCu-Ti, MCu-Ti, HCu-Ti) for 24 hours (left column), for 48 hours (column in the middle) and for 96 hours (right column).

2. Supplementary Tables

Table S1. Applied operating parameters for the surface modification of the poly(lactide-co-glycolide) (PLGA) scaffolds by magnetron co-sputtering of copper (Cu) and titanium (Ti).

Sample	Discharge Power (W)		Voltage (V)		Current (A)		Sputtering Time (min)
	Cu	Ti	Cu	Ti	Cu	Ti	
LCu-Ti	70	750	430	500	0.15	1.5	26.6
MCu-Ti	130	500	430	450	0.30	1.1	29.3
HCu-Ti	200	500	430	450	0.45	1.1	19.5

Table S2. Released amount of copper ions from the surface-modified PLGA scaffold samples (LCu-Ti, MCu-Ti, HCu-Ti).

Time of PLGA samples interaction with water (Days)	Cu Release ($mg \cdot L^{-1}$)		
	LCu-Ti	MCu-Ti	HCu-Ti
30 minutes (0 Days)	0.02 ± 0.00	0.04 ± 0.02	0.06 ± 0.01
1	0.04 ± 0.01	0.16 ± 0.05	0.53 ± 0.16
3	0.06 ± 0.02	0.66 ± 0.20	0.64 ± 0.19
7	0.06 ± 0.02	1.04 ± 0.31	1.50 ± 0.40
14	0.10 ± 0.03	1.14 ± 0.34	2.41 ± 0.72
28	0.08 ± 0.03	1.59 ± 0.48	2.03 ± 0.61

Table S3. Release rate of copper ions from the surface-modified PLGA scaffold samples (LCu-Ti, MCu-Ti, HCu-Ti).

Time of PLGA samples interaction with water (Days)	Cu Release Rate ($mg \cdot L^{-1} \cdot d^{-1}$)		
	LCu-Ti	MCu-Ti	HCu-Ti
30 minutes (0 Days)	0.96 ± 0.20	2.02 ± 0.82	2.69 ± 0.62
1	0.02 ± 0.00	0.12 ± 0.04	0.47 ± 0.14
3	0.01 ± 0.00	0.25 ± 0.08	0.06 ± 0.02
7	–	0.10 ± 0.03	0.22 ± 0.06
14	–	0.01 ± 0.00	0.13 ± 0.04
28	–	–	–
